# PBMC transcriptome profiles identifies potential candidate genes and functional networks controlling the innate and the adaptive immune response to PRRSV vaccine in Pietrain pig

**DOI:** 10.1371/journal.pone.0171828

**Published:** 2017-03-09

**Authors:** Md. Aminul Islam, Christine Große-Brinkhaus, Maren Julia Pröll, Muhammad Jasim Uddin, Sharmin Aqter Rony, Dawit Tesfaye, Ernst Tholen, Michael Hoelker, Karl Schellander, Christiane Neuhoff

**Affiliations:** 1 Department of Animal Breeding and Husbandry, Institute of Animal Science, University of Bonn, Endenicher Allee 15, 53115 Bonn, Germany; 2 Department of Medicine, Faculty of Veterinary Science, Bangladesh Agricultural University, Mymensingh-2202, Bangladesh; 3 School of Veterinary Science, University of Queensland, Gatton Campus, QLD 4343, Australia; 4 Teaching and Research Station on Frankenfrost, Faculty of Agriculture, University of Bonn, Königswinter, Germany; University of Illinois, UNITED STATES

## Abstract

The porcine reproductive and respiratory syndrome (PRRS) is a devastating viral disease affecting swine production, health and welfare throughout the world. A synergistic action of the innate and the adaptive immune system of the host is essential for mounting a durable protective immunity through vaccination. Therefore, the current study aimed to investigate the transcriptome profiles of peripheral blood mononuclear cells (PBMCs) to characterize the innate and the adaptive immune response to PRRS Virus (PRRSV) vaccination in Pietrain pigs. The Affymetrix gene chip porcine gene 1.0 ST array was used for the transcriptome profiling of PBMCs collected at immediately before (D0), at one (D1) and 28 days (D28) post PRRSV vaccination with three biological replications. With FDR <0.05 and log2 fold change ±1.5 as cutoff criteria, 295 and 115 transcripts were found to be differentially expressed in PBMCs during the stage of innate and adaptive response, respectively. The microarray expression results were technically validated by qRT-PCR. The gene ontology terms such as viral life cycle, regulation of lymphocyte activation, cytokine activity and inflammatory response were enriched during the innate immunity; cytolysis, T cell mediated cytotoxicity, immunoglobulin production were enriched during adaptive immunity to PRRSV vaccination. Significant enrichment of cytokine-cytokine receptor interaction, signaling by interleukins, signaling by the B cell receptor (BCR), viral mRNA translation, IFN-gamma pathway and AP-1 transcription factor network pathways were indicating the involvement of altered genes in the antiviral defense. Network analysis revealed that four network modules were functionally involved with the transcriptional network of innate immunity, and five modules were linked to adaptive immunity in PBMCs. The innate immune transcriptional network was found to be regulated by LCK, STAT3, ATP5B, UBB and RSP17. While TGFß1, IL7R, RAD21, SP1 and GZMB are likely to be predictive for the adaptive immune transcriptional response to PRRSV vaccine in PBMCs. Results of the current immunogenomics study advances our understanding of PRRS in term of host-vaccine interaction, and thereby contribute to design a rationale for disease control strategy.

## Introduction

Porcine reproductive and respiratory syndrome virus (PRRSV) is the causative agent of an economically important swine disease, which is clinically characterized by reproductive failure in pregnant sows and respiratory disorder in young pigs [1]. The PRRSV is a positive-sense, single-stranded RNA virus having two distinct genotypes namely European and North American. In swine, the common symptoms to PRRSV infection has been characterized by prolonged viremia, a deficient induction of innate immunity along with weak and delayed development of neutralizing antibodies [2, 3] which are the major hurdle for control of porcine reproductive and respiratory syndrome (PRRS). Therefore, elucidating the main genomic factors involved in developing protective immune response to PRRSV vaccination is of utmost importance.

The modified live virus (MLV) based vaccination has commonly been practiced as one of the primary and economic tools for swine herd immunization against PRRS [4]. The MLV-PRRS vaccination can provide protection at least against reinfection with homologous PRRSV isolates and minimizes the clinical outbreaks [5]. However, the molecular pathways and functional networks involved during the acqusition of immunity to PRRSV via vaccination have not yet been entirely elucidated. It is conceivable that the host response may differ to vaccine antigen to some extend from that of virulent infectious virus. The PRRSV infection has a predilection for the cells of mononuclear phagocytic lineage, like pulmonary alveolar macrophages and blood monocytes [6]. The virulent PRRSV infection causes depletion of immune cells through cytophathic replication preferably within the alveolar macrophage. While the attenuated virus strain used as vaccine is likely unable to cause cytopathic effects, it is able to sensitize the blood macrophage in the same way as virulent virus and induces immune response afterwards [7, 8]. Moreover, the quality of immunity derived from natural PRRSV infection seemed not ideal for the implementation in the vaccine development programs [9] that provoked the molecular characterization of host-vaccine interaction.

The host immune response to vaccination is comprised of a complex interplay between components of the innate and the adaptive immune system [10]. Innate immunity is the initial body defense against invading pathogen, typically occurs within hours to few days of exposure through recognition of conserved epitopes followed by triggering a proinflammatory response [11]. While the adaptive immunity represents the neutralizing antibody response usually developed at 2–4 weeks following antigenic stimulation in a pathogen-specific manner through generating the immunological memory [12]. Antibodies are the essential vaccine induced immune effectors produced by B lymphocytes, and are capable of binding specifically to a pathogen or antigen. Other potential effectors are cytotoxic CD8^+^ T lymphocytes (CTLs) that may limit the spread of infectious agents by recognizing and killing infected cells or secreting specific antiviral cytokines. The live attenuated viral vaccine elicits the recruitment of antigen specific CD4^+^ Th cells in the circulation which lead to induce both higher affinity antibody and immune memory, known as T dependent antibody responses [13]. The balanced host immunocompetence with cell-mediated (Th1) and humoral (Th2) immune responses is a proposed selection goal for general disease resistance [14]. Thus, identification of transcriptome signatures for the innate and the adaptive response to PRRSV vaccination might contribute to design a rationale husbandry and breeding scheme for sustainable PRRS control.

The host immune response to PRRSV has been studied through global transcriptome profiling mainly of lung tissue [15, 16] and pulmonary alveolar macrophage [17–20] with either in-vitro or in-vivo PRRSV infection, whereas reports on blood-based transcriptional response to vaccination are sparse. Considering the sampling convenience, time entailed, and animal welfare issues, the peripheral blood samples are much preferred to respiratory tissues/cells for evaluating the host immune responses to PRRSV vaccination. Moreover, unlike the sampling of pulmonary alveolar macrophage, repeated blood sampling is possible from the same individual during the course of immune responses, which is especially useful in controlling the baseline variation [21]. Furthermore, blood based genomic biomarkers can significantly advance the herd health management for PRRS by, for example, allowing the rapid and early prediction of host immunocompetence developed from vaccination [22]. Among the fractions of whole blood, the white blood cells transcriptome profile assume to reflect the transcriptomes of other porcine immune cells, likely what has been demonstrated in case of human [23].

Peripheral blood mononuclear cells (PBMCs), a subset of while blood cells have been proved to be a suitable model for characterizing the host immune response to vaccines in human (reviewed in [24]). The porcine PBMCs have also been studied by some authors for the evaluation of immune response to PRRSV in pigs [8, 25, 26]. However, those studies were mostly focused on expression profiling of selected candidate genes using the in-vitro model. The current study employed an in-vivo PBMCs model to characterize the innate as well as the adaptive immune response to PRRSV vaccine through a global transcriptome approach. In a recent study, we observed that the highest transcriptional response of PRRSV vaccine during first three days occured at 24 h after vaccination; we also observed a plateaued plasma antibody response to PRRSV vaccine at 28 days after primary vaccination [27]. Therefore, we extended our aim herein to investigate the PBMCs transcriptome profiles at day 1, and day 28 post PRRSV vaccination to characterize functional networks associated with the innate and the adaptive immune response to PRRSV in Pietrain pig, respectively.

## Materials and methods

### Ethics statements

The research proposal was approved by the Veterinary and Food Inspection Office, Siegburg, Germany (ref. 39600305-547/15). The whole in-vivo experiment was conducted according to the institutional guidelines and animal husbandry regulations of Germany [28]. The blood sampling protocol was approved by the State Agency for Nature, Environment and Consumer Protection, North Rhine-Westphalia, Germany (permission nr. 84-02.05.04.14.027).

### Vaccination and blood sampling

Littermate piglets of two purebred Pietrain sows were housed in the teaching and research station at Frankenforst, University of Bonn, Germany. Six clinically healthy female piglets from two sows, free from history respiratory diseases were included in this study. Piglets were immunized with the commercially available modified live PRRSV vaccine of European strain (Porcilis^®^ PRRS, MSD Animal Health, Germany) through intramuscular injection of primary dose at day 28, and booster dose at day 56 of their age. About 7 mL anti-coagulated venous blood samples were collected from all piglets repeatedly at day 7, 28, 29, 42, 56 and day 70 of age. The blood samples from all animals at all the time points were screened by ELISA for monitoring the PRRSV-specific antibody responses. However, for microarray study, the blood samples collected at just before (D0), and one day (D1) and 28 days (D28) relative to the primary vaccination were used with three biological replications.

### Isolation of PBMCs and plasma

The PBMCs were isolated from the whole blood by the density gradient centrifugation with Ficoll-Paque (Histopaque^®^-1077; Sigma-Aldrich, Munich, Germany) according to the protocol described by Uddin et al. [29]. In brief, whole blood were diluted at the ratio of 1:1 with phosphate buffered saline (PBS) and carefully layered over 8 mL of Histopaque solution previously kept in a 50 mL conical tube. Then the tubes were centrifuged at 1,250x g for 30 min at room temperature. After centrifugation, plasma was aspirated from the upper most layers and kept at −20°C until used. PBMCs preparation was carefully aspirated and treated with RBC lysis buffer (Invitrogen, Darmstadt, Germany) to eliminate erythrocytes. Finally, PBMCs were washed twice with PBS and harvested as pellet.

### Monitoring of plasma antibody response

To monitor the PRRSV-specific antibody titre, the plasma samples from all animals collected at day 7, 28, 42, 56 and 70 of age were screened by ELISA (PRRSV-AK screening, Synlab Vet GmbH, Augsburg, Germany) according to manufacturer’s protocol. The optical density (OD) of each well was measured at 650 nm using the Bio-Rad 680 microplate reader. The presence or absence of PRRSV antibody was determined by calculating the sample to positive (S/P) ratio. The S/P ratio was calculated according to the equation described in our previous study [27]. The samples were considered to be positive for PRRSV antibody if the S/P ratio was more than 0.4. One-way repeated measures ANOVA was performed to compare the mean antibody titre obtained at different time points relative to vaccination using the GraphPad Prism v.5 [30]. The *P*-value of <0.05 was considered for significance.

### RNA extraction and microarray hybridization

Total RNA was extracted from PBMCs using the miRNeasy mini kit (P/N 217004, Qiagen, Hilden, Germany) according to the manufacturer’s protocol along with on column DNase treatment (P/N 79254, Qiagen, Hilden, Germany). The RNA integrity was checked by micro capillary electrophoresis on an Agilent 2100 Bioanalyser with RNA 6000 Nanochip kit (Agilent Technologies, Waghäusel—Wiesental, Germany). The total RNA from three individual piglets collected at D0, D1 and D28 time points were used for preparing the target probes for nine microarrays. About 100 ng of total RNA were processed to synthesize the biotin-labeled sense strand cDNA (ss-cDNA) probes using the GeneChip WT PLUS Reagent kit (P/N 902281; Affymetrix Inc., Santa Clara, CA, USA) according to the manufacturer’s protocol. About 130 μL of biotinylated ss-cDNA probes were injected into the GeneChip Porcine Gene 1.0 ST array of 81/4 format (P/N 901976, Affymetrix) and incubated for 16 hours in a hybridization oven (GeneChip Hybridization oven 640; Affymetrix) at 45°C with 60 rpm for hybridization. The hybridized chips were stained and washed in a fluidics station (GeneChip Fluidics Station 450; Affymetrix) and scanned by Affymetrix GeneChip scanner 3000 7G. The image data were evaluated using Affymetrix Genechip command consol (AGCC) software and the intensity data were exported into .CEL file format. The MIAME (minimum information about microarray experiment) complaint raw data have been submitted into the gene expression omnibus (GEO) database with the accession code GSE84516.

### Microarray data processing and statistical analysis

The normalization and background correction of microarray dataset were performed using the ‘oligo’ Bioconductor package [31] implemented in R project software (v3.1.2) [32]. The RMA (Robust Multi-array Average) based quantile normalization (log2) of microarray data was performed at the transcript level. Probe to gene transcript annotation was performed with recent Affymetrix annotation file [33]. Gene annotations were extended by their orthologous human gene symbol as well as ensembl identifiers using the BioDBnet.org tool (http://biodbnet.abcc.ncifcrf.gov/). To explore the transcriptional modifications in response to vaccination, differential gene expression analysis was performed using the linear analysis of microarray technique from the ‘limma’ package [34] with empirical Bayes adjustment to the variance, followed by Benjamini and Hochberg (BH) correction for multiple testing [35]. Two pairwise contrasts such as D1 *vs*. D0 and D28 *vs*. D0 were considered for differential gene expression associated with the innate and the adaptive immune response, respectively. The false discovery rate (FDR) of <0.05 and log2 fold-change either >1.5 or <−1.5 were considered as threshold for differential expression of genes.

### Functional analysis of differentially expressed genes

For biological interpretation of the transcriptome dataset, the significantly over-represented gene ontology (GO) terms, biological pathways, and transcription factor binding sites (TFBS) were explored with the InnateDB pathway analysis tool [36]. InnateDB platform implements the hypergeometric algorithm with the Benjamini-Hochberg (BH) multiple test correction method for overrepresentation analysis. For this analysis, the list of ensembl gene identifiers was uploaded in InnateDB web and performed the over-representation analysis. GO, pathways and TFBS were considered significantly over-represented with an FDR <0.05.

### Cell-type enrichment analysis of differentially expressed genes

To get an overview on which subtypes of PBMCs contribute in vaccine induced differential gene expression, DEGs were anlyzed using the CTen web-portal. The CTen (**c**ell **t**ype **en**richment) is an online bioinformatics tool for identifying enriched cell types in heterogeneous microarray data [37]. This tool implements a highly expressed, cell specific (HECS) gene database comprises of 10,058 genes of human and mouse origin. For this analysis, human orthologus symbol of differentially expressed genes were uploaded and compared with human HECS database. The significance of enrichment was determined using the one-tailed Fisher exact test and P values were adjusted with Benjamini-Hochberg (BH) method across all cell types. The enrichment score estimated as −log10 of the BH-adjusted P value and created the color-coated output figures indicating this enrichment score.

### Network analysis of differentially expressed genes

The ensembl orthologous identifiers of the differentially expressed genes were uploaded into the NetworkAnalayst tool [38] to construct the weighted network based on Walktrap algorithm by taking the first order interacts (direct interaction of seed genes). For high-performance visualization, the network size was adjusted for maximum of 500 nodes and 1200 edges using the ‘trim’ function of the tool. Two topological measures such as degree (number of connections it has to other nodes) and betweeness centrality (number of shortest paths going through the node) were taken into account for detecting highly interconnected hub of the network. Nodes having higher degree and betweenness values are the potential network hubs regulating cellular signal trafficking. The weighted network based module detection was performed to stratify the interconnected genes of similar biological function. For statistical significance, p value of a given network module was calculated using a Wilcoxon rank-sum test of the “internal” (edges within in a module) and “external” (edges connecting the nodes of other modules) degrees. Modules having more internal than external edges were like to be significant. Finally, the in-situ functional enrichment of the modules was performed based on REACTOME.db pathway database.

### RT-qPCR validation

Four selected differentially expressed genes those are known to be involved in immune response, were quantified by RT-qPCR for the technical validation of microarray results (Table 1). Primers were designed based on an open source primer designing software Primer3 [39]. The reverse transcription was performed using First Strand cDNA Synthesis Kit (P/N K1612, Thermo Scientific, Co.). The RT-qPCR reaction was set up taking 1.0 μl of cDNA template, 8.0 μl of deionized RNase free water, 0.5 μl of upstream and downstream primers, and 10 μl iTaq^™^ Universal SYBR^®^ Green Supermix (Bio-Rad laboratories GmbH, Germany) in a total volume of 20 μl. All reactions were amplified in duplicate by the StepOnePlus^™^ Real-Time PCR System (Applied Biosystems^®^, Darmstadt, Germany) with thermal cycling conditions of 95°C for 3 min, 95°C for 15 sec, 60°C for 45 sec (40 cycles); 95°C for 15 sec, 62°C for 1 min, 95°C for 15 sec. The delta Ct (ΔCt) [ΔCt = Ct target—Ct house keeping genes] values were calculated as the difference between target gene and the average of reference genes (GAPDH and ACTB), and the relative expression was calculated as 2(-ΔCt) [40]. The correlation between RT-qPCR and microarray results was estimated through Pearson’s correlation test using the GraphPad Prism^®^ v.5 [30].

**Table 1:** Sequences of the primers used for RT-qPCR validation of microarray results.

Accession	Symbol	Sequence (5′-3′)	Ann. Temp. (°C)	Size(bp)
*BNM*_213948.1	*IFNG*	*F*: *AGCTCCCAGAAACTGAACGA* *R*: *AGGGTTCAAAGCATGAATGG*	60	225
*NM*_214015	TGFß1	*F*: *ACTACTACGCCAAGGAGGTCA* *R*: *TCTGCCCGAGAGAGCAATAC*	60	157
*NM*_213997.1	*IL8*	*F*: *TAGGACCAGAGCCAGGAAGA* *R*: *CAGTGGGGTCCACTCTCAAT*	60	174
*NM*_214041.1	*IL10*	*F*: *GTGGAGGAGGTGAAGAGTGCC* *R*: *GAGGTACAGCAGGGTTTCCCA*	60	266
*HQ*013301	*GAPDH*	*F*: *GCTGGTGCTGAGTATGTCGT* *R*: *AAGCAGTTGGTGGTACAGG*	56	124
*XM*_003124280.3	*ACTB*	*F*: *AAGGACCTCTACGCCAACAC* *R*: *CTGGCTGATCCACATCTGCT*	57	110

GAPDH and ACTB were used as reference for normalization; Ann. Temp.: Annealing temperature; bp: base pair; F: Forward; R: Reverse.

## Results

### Antibody response to PRRSV vaccine

**Fig 1:**
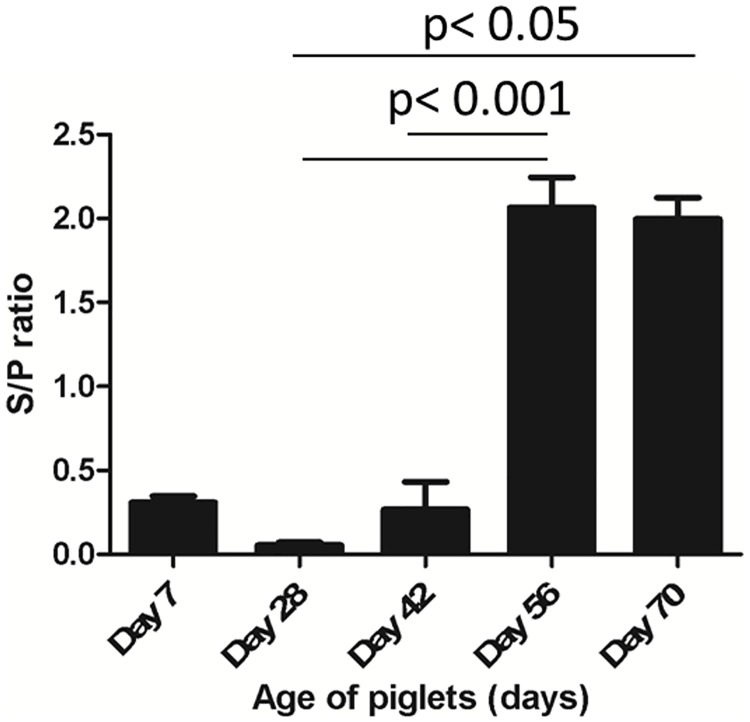
PRRSV specific antibody response. The figure illustrates the reactivity of maternally derived antibody, and vaccine derived antibody to PRRSV in plasma detected by ELISA. The optical density (OD) values in the Y-axis represents sample to positive (S/P) ratio, and a S/P value of 0.4 was considered as threshold for positivity of antibody response. Values in X-axis represents the piglet ages at which blood samples were evaluated. Blood sampling at day 28 and day 56 were performed right before the primary and the booster vaccination, respectively (indicated by asterisk).

The PRRSV-specific antibody titre in the blood plasma at day 7, 28, 42, 56 and day 70 of age was measured by ELISA to evaluate the antibody response derived from maternal origin and/ or from vaccination. It revealed that piglets were negative for maternally derived antibody at the time of primary vaccination considering an optical density (OD) value of 0.4 as threshold (Fig 1). The PRRSV vaccine-specific antibody response was appeared at 14 days (day 42 of age) post priming followed by a significantly (*P* <0.001) increased titre at 28 days (day 56 of age) post priming. A significantly (*P* <0.05) high level of antibody response were continued over the period of four to six weeks of primary vaccination.

### Transcriptome profiling of PBMCs following PRRSV vaccination

In order to investigate the host transcriptional response to PRRSV vaccine, we employed the Affymetrix GeneChip Porcine Gene 1.0 ST Array for the whole transcriptome profiling of PBMCs collected immediately before (D0), and at one (D1) and 28 days (D28) post vaccination in Pietrain pigs with three biological replicates. First, the normalized gene probe sets were filtered to eliminate those with very low expression summary values and low variability across the samples. After filtering, the normalized expression data yielded a total of 14,212 gene transcripts to be expressed in PBMCs following vaccination. While the array chip used in this study were embedded with probe sets of 19,218 known genes in total.

### PBMCs transcriptome alteration associated with innate and adaptive immunity to PRRSV vaccine

**Fig 2:**
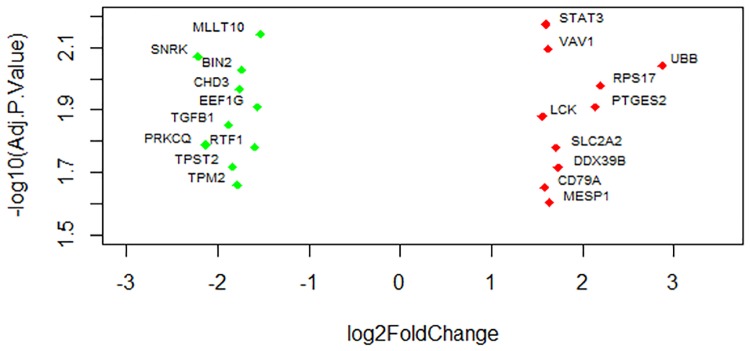
Volcano plot showing the most significantly altered genes at day one post vaccination. The picture demonstrates the range of fold changes of most significantly altered transcripts in connection to innate immune response in PBMCs.

Transcripts were considered to be differentially expressed having the log fold change of >1.5 or <1.5 and false discovery rate (FDR) of <0.05. Imposing this cutoff, a total of 295 transcripts were found to be differentially expressed in PBMCs at day one post vaccination compared to control. The expression level of 65 genes including STAT3, LCK, UBB, VAV1, RSP17, SLC2A2, PTGES2 and MESP1 were upregulated and 230 genes including TGFß1, RTF1, BIN2, TPST2, SNRK and PRKCQ were downregulated (Table 2). The range of log fold change of differentially expressed genes was between −4.461 and 3.46. The extend of fold change of most significantly (FDR sorted top ten up- and down- regulated) altered genes associated with innate immunity are presented in volcanoplot (Fig 2). The complete list of differentially expressed genes is provided in S1 Table.

**Table 2:** Number of differentially expressed genes in PBMCs following PRRSV vaccination.

Types	Number of genes	
	Innate immunity (D1 *v*s. D0)	Adaptive immunity (D28 *v*s. D0)
Up regulated	65	37
Down regulated	230	78
**Total**	**295**	**115**

**Fig 3:**
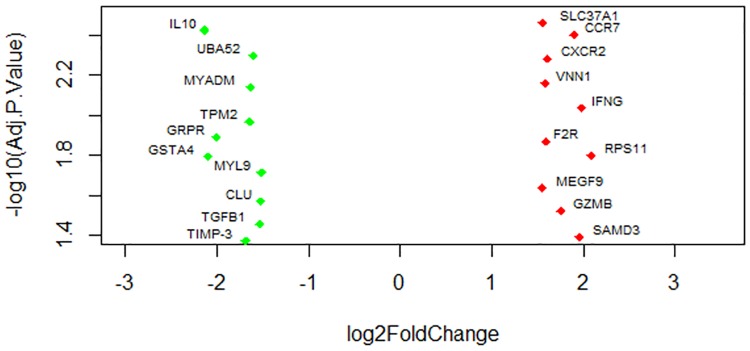
Volcano plot showing the most significantly altered genes at day 28 post vaccination. The picture demonstates the range of fold changes of most significantly altered transcripts in connection to adaptive immune response in PBMCs.

At day 28 post vaccination, a total of 115 genes were identified as differentially expressed, with 37 being upregulated and 78 being down regulated under the same threshold as above (Table 2). The volcano plot (Fig 3) of the FDR sorted top ten altered genes indicated that CXCR2, IFNG, SMAD3, VNN1, F2R and GZMB genes were most significantly upregulated and IL10, MYL9, TPM2, GSTA4, CLU and TGFß1 were down regulated in PBMCs following PRRSV vaccination, among the list of differentillay expressed genes (DEGs). The range of log fold change of DEGs was between −2.51 and 4.50. A complete list of the differentially expressed genes in PBMCs after 28 days of PRRSV vaccination is provided in S2 Table.

**Fig 4:**
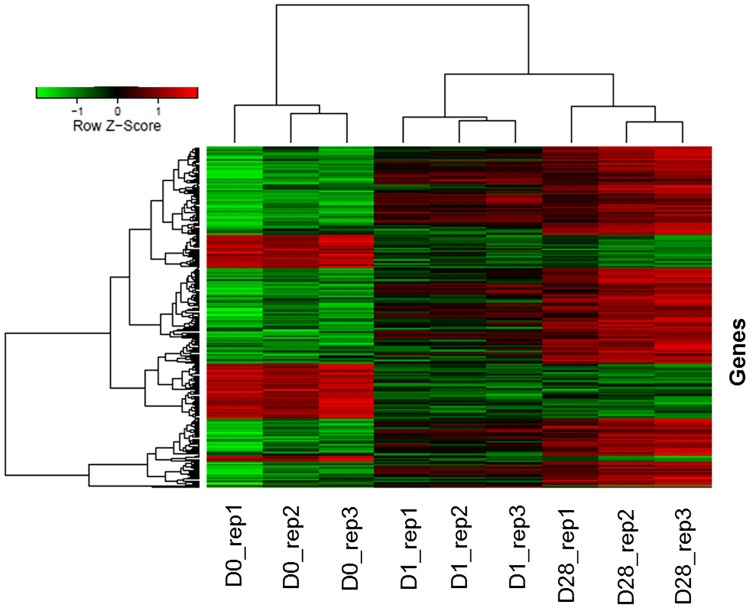
Hierarchical heat map showing the expression dynamics of DEGs. Normalized log_2_ transformed values as determined by Affymetrix GeneChip^®^ porcine gene 1.0 ST array in PBMCs of Pietrain pigs collected at D0, D1 and D28 of PRRSV vaccination. Each column represents one pig, three replicates at each time point, each horizontal line refers to one gene. The cutoff value of log fold change as >1.5 or <−1.5 and false discovery rate <0.01 was considered.

The hierchialcal heatmap demonstrated the visual summary of the dynamic changes in the transcriptional response to PRRSV vaccine at two time points reflecting a gradual upregulation of differentially expressed transcripts with two major clusters (Fig 4). Samples clustering revealed two superior clusters, one for pre vaccinated and another for post vaccinated samples. The replicates of each sampling time points are clustered together indicated a low individual variation on the vaccine induced gene expression.

### GO terms and pathways enriched by Differentially Expressed Genes (DEGs)

The Gene Ontology analysis revealed that vaccine induced differentially expressed genes are involved in the process of active cellular process (Table 3), including T cell response (eg, GO:0050852, GO:0051249, GO:00190058), cellular protein metabolism (eg, GO:0044267, GO:0030162, GO:0001948, GO:0005840), gene expression (eg, GO:0010467, GO:0006412, GO:0050852) and regulation of apoptosis (eg, GO:0043065) during the early stage of vaccine exposure. On the other hand, DEGs observed at 28 days post vaccination are involved with enrichment of GO terms including B cell proliferation (eg, GO:0042100) inflammatory response (eg, GO: 0006954), MHC class II biosynthesis (eg, GO:0045348), gene expression (eg, GO: 0010628), antigen processing and presentation (eg, GO:0019882). Overall, there were significantly altered transcripts participating in cellular activation and differentiation, protein metabolism and gene expression (Table 3).

**Table 3:** Significantly enriched Gene Ontology (GO) terms involved with DEGs.

Contrasts	ID	Description	Category	Genes*^a^*	Adj. P
**D1 *v*s. D0**	GO:0019058	Viral life cycle	BP	6	0.0067
	GO:0006412	Translation	BP	7	0.0154
	GO:0051249	Regulation of lymphocyte activation	BP	6	0.0259
	GO:0010467	Gene expression	BP	19	0.0269
	GO:0070062	Extracellular vesicular exosome	CC	8	0.0273
	GO:0006200	ATP catabolic process	BP	11	0.0299
	GO:0050852	T cell receptor signaling pathway	BP	6	0.0301
	GO:0030162	Regulation of proteolysis	BP	13	0.0310
	GO:0005840	Ribosome	CC	17	0.0323
	GO:0022857	Transmembrane transporter activity	MF	12	0.0327
	GO:0045747	Positive regulation of Notch signaling pathway	BP	9	0.0336
	GO:0043065	Positive regulation of apoptotic process	BP	6	0.0339
	GO:0044267	Cellular protein metabolic process	BP	16	0.0343
	GO:0001948	Glycoprotein binding	MF	15	0.0343
**D28 *v*s. D0**	GO:0005515	Protein binding	MF	19	0.0332
	GO:0005886	Plasma membrane	CC	7	0.0317
	GO:0005576	Extracellular region	CC	8	0.0242
	GO:0006954	Inflammatory response	BP	6	0.0043
	GO:0008284	Positive regulation of cell proliferation	BP	7	0.0143
	GO:0010628	Positive regulation of gene expression	BP	14	0.0198
	GO:0006915	Apoptotic process	BP	6	0.0341
	GO:0042100	B cell proliferation	BP	5	0.0053
	GO:0009615	Response to virus	BP	5	0.0191
	GO:0005125	Cytokine activity	MF	6	0.0224
	GO:0043123	Positive regulation of NFkB signaling	BP	6	0.0240
	GO:0007166	Cell surface receptor signaling pathway	BP	9	0.0299
	GO:0045348	Positive regulation of MHC class II biosynthesis	BP	5	0.0068
	GO:0009611	Response to wounding	BP	6	0.0256
	GO:0019882	Antigen processing and presentation	BP	6	0.0261

*^a^*: Number of genes involved in corresponding GO terms but one gene may appear in multiple terms, BP: Biological process, CC: Cellular component, MF: Molecular function, Adj.P: P values adjusted for multiple test correction method

**Fig 5:**
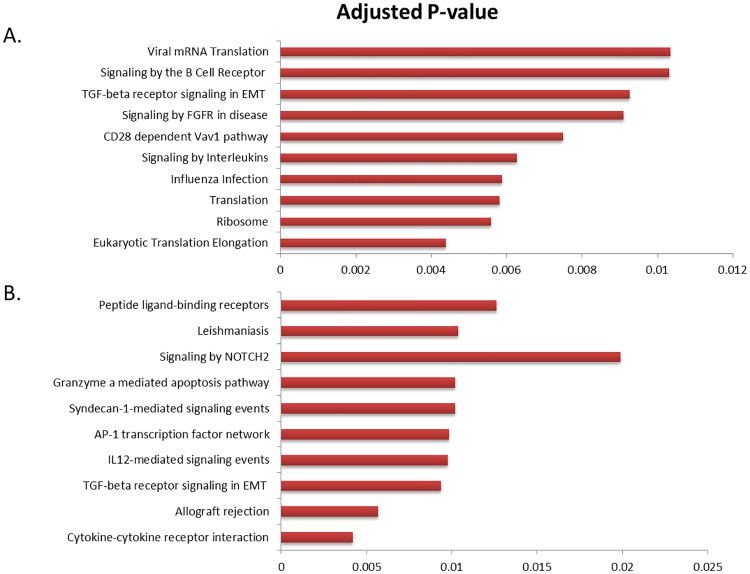
Significantly enriched pathways by DEGs. Significantly enriched pathways in PBMCs at 1 (A), and 28 (B) days post PRRSV vaccination in pig.

Pathways enrichment analysis revealed the involvement of several immune response pathways with PRRSV vaccine induced gene expression in PBMCs including signaling by B cell receptor, CD28 dependent VAV1 signaling, signaling by interleukins, influenza infection and TGFß signaling pathways at one day after vaccination (Fig 5A). Signaling by NOTCH2, peptide-ligand binding receptor, Granzyme mediated apoptosis pathway, AP-1 transcription factor network and TGFß signaling pathways were significantly enriched at 28 day post vaccination(Fig 5B).

### Transcription factor binding sites of DEGs

**Fig 6:**
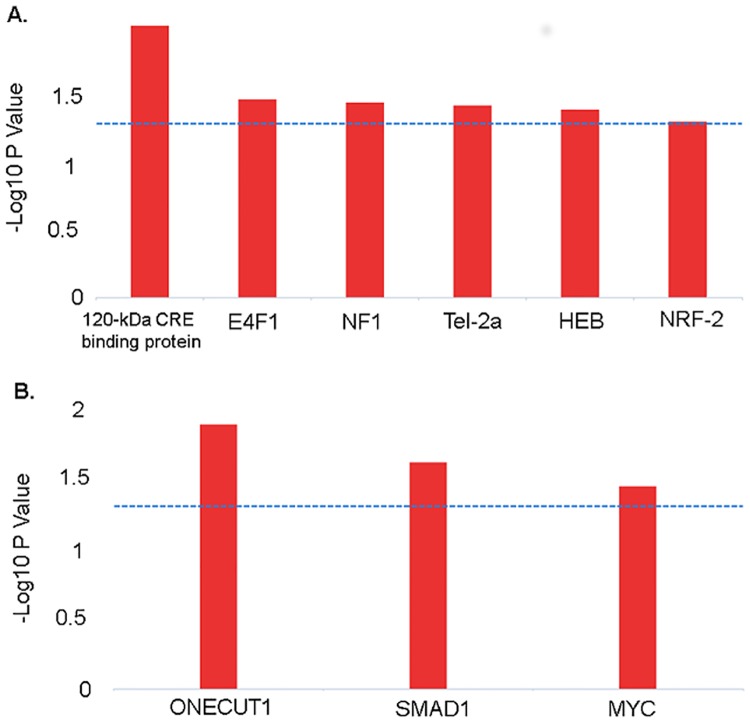
Transcription factors binding sites of DEGs. The figure depicts the TFBS of the genes showing differential expression in PBMCs at one day after vaccination (A), and 28 days after vaccination (B). Blue dotted lines indicate the threshold (-log10 P value of 1.3) for statistical significance.

We explored the involvement of transcription factors in the differential gene expression in vaccinated PBMCs using the InnateDB database. The transcription factor binding site (TFBS) analysis revealed that 120-kDa CRA-binding protein, E4F10, NF1, Tel-2a, HEB and NRF-2 genes have the transcription factor binding sites which are likely contributing to PBMCs transcriptome alteration at early stage of PRRSV vaccination (Fig 6A). The TFBS analysis also revealed that ONECUT1, SMAD1 and MYC have the transcription factor binding sites regulating transcriptional machinery for inducing adaptive immune response in PBMCs (Fig 6B). The PRRSV vaccine induced differentially expressed genes that are predicted to be regulated by the transcription factors are presented in Table 4.

**Table 4:** The known target genes bound by transcription factors identified in PBMCs after PRRSV vaccination in pigs.

Transcription factors	Potential target genes	P-value
120-kDa CRE-binding protein	DDX39B, RAP2A, RPS11, SERPINC1, STAT3 and TRPC4AP	0.00928
E4F1	DUSP1, RAP2A, RPS11, STAT3 and UBL5	0.03250
NF-1	DAPL1, DDX39B, EN2, LCK, RAP2A, SERPINC1, STAT3, TRPC4AP, UBB and UQCRH	0.03421
Tel-2a	LCK, RPS11, TRPC4AP and UBL5	0.03611
HEB	DAPL1 and TRPC4AP	0.03872
NRF-2	LCK, RPS13, TRPC4AP and UBL5	0.04773
ONECUT1	ANGPT2, DGKA, F2R, GZMB and TGFB1	0.01281
SMAD1	GZMB, IL7R, RSAD2, SMAD3, SCL37A1 and VNN1	0.02389
MYC	ANGPT2, IFNG, PLAC8 and TGFB1	0.03546

The target genes presented here are only the common genes of DEGs in PBMCs after PRRSV vaccination

### Cell-type specific pattern of gene expression

**Fig 7:**
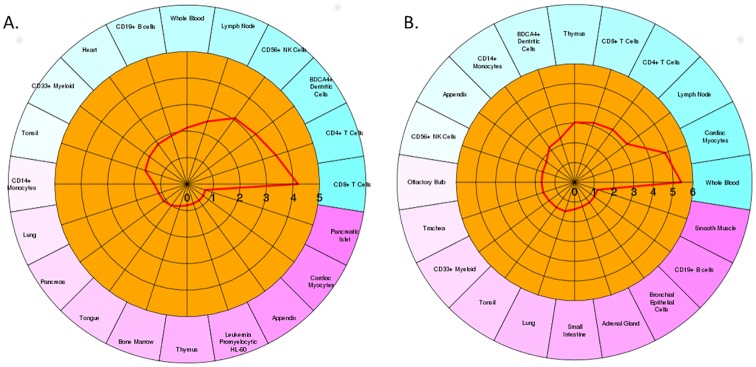
Circular plot showing the cell-type enrichment of DEGs. The figure depicts the cell-type specific enrichment of differentially expressed genes in PBMCs at one day after vaccination (A), and 28 days after vaccination (B). Red bold lines intersecting the cell types indicate the enrichment score (-log 10 adj. P-value) intersecting the cell type. The enrichment score cutoff of 2 or more was considered for statistical significance.

To predict the specific cell-type contribution on vaccine induced differential genes expression in PBMCs, we tested the list of DEGs using an enrichment algorithm implemented in CTen web-portal. It revealed that differential expression of transcriptomes at early vaccine exposure was found to be significantly contributed by innate immune cell types including CD56^+^ NK cells, BDCA4^+^ dendritic cells, CD4^+^ T cells and CD8^+^ T cells (Fig 7A). While differentially expressed genes at 28 days post vaccination were of multiple cell-type origin including CD14^+^ monocytes, BDCA4^+^ dendritic cells, thymus, CD8^+^ T cells, CD4^+^ T cells, lymphnodes and whole blood (Fig 7B). Some cell types were mutually contributing to differential gene expression in both time points. NK cells was the enriched cell type associated with innate immunity but not with adaptive immunity in PBMCs. On the other hand, thymus, CD14^+^ and whole blood sample cell types were enriched in connection to adaptive immune response, but not during the stage of innate immunity to PRRSV vaccination in pig.

### Functional network of innate immune transcripts

**Fig 8:**
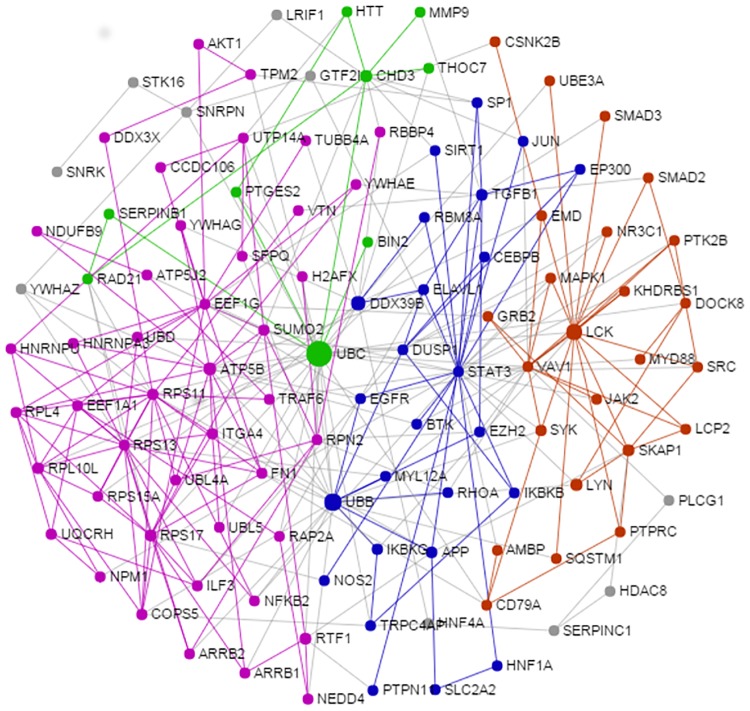
Network of PRRSV vaccine induced innate immune transcriptomes in PBMCs. The figure demonstrates the interconnected network of PRRSV vaccine induced differentially expressed genes in PBMCs at one day after PRRSV vaccination compared to before vaccination in Pietrain pigs. Each circle indicates the node or member genes of the network. The diameter of the circle corresponds to the values of two centrality measures (degree and betweenness). The larger diameter indicates the higher potential of the nodes to be the hub genes of the network. The network modules with corresponding genes are indicated by different colors (purple: IM0, blue: IM1, pink: IM2 and green: IM3).

The simplified network of PRRSV vaccine induced innate immune transcripts in PBMCs is presented in (Fig 8). The network topology analysis showed that UBD, LCK, STAT3, ATP5B, RPS11, RPS13, RPS17 and EEF1G are the highly interconnected hubs of the network. The majority of core genes of the network were overexpressed in PBMCs following PRRSV vaccination indicated the upregulation of their underlying function. The network module analysis revealed that differentially expressed genes were clustered in four modules (IM0, IM1, IM2 and IM3) indicated by four different colors (Fig 8).

The purple module (IM0) contains the genes (LCK, SKAP1, MyD88, MAPK14, VAV1, JAK2, SRC, CD79A, PTPRC, AMBP, DOCK8, PTK2B, SMAD3, CSNK2B, UBE3A, KHDRBS1) and is functionally linked to various innate immune response functions such as signaling by interleukins, cytokine signaling in immune system, CD28 co-stimulation, antigen activates B cell receptor leading to generation of second messengers, signaling by SCF-KIT, Fc gamma receptor (FCGR) dependent phagocytosis, interleukin-1 signaling, integrin cell surface interactions, innate immune system, signal transduction, TRAF6 mediated induction of NFkB and MAP kinases upon TLR7/8 or 9 activation. The blue module (IM1) containing genes such as STAT3, TGFß1, APP, DUSP1, ELAVL1DDX39B, MYL12A, EP300, IKBKB, IKBKG, RBM8A, SP1, EGFR, TRPC4A and CEBPB. These genes altogether involved in biological functions like MyD88:Mal cascade initiated on plasma membrane, toll-like receptors cascades, signaling by interleukins, NFkB activation by phosphorylation and activation of IKKs complex, cytokine signaling in immune system, signaling by the B cell receptor (BCR) and activation of NFkB in B cells. The pink module (IM2) containing genes such ATP5B, RSP11, RSP13, RSP17, UTP14A, EEF1G, EEF1A1, SUMO2, ITGA4, TRAF6, FN1, RPN2, RPL4, RPL10L, COPS5, HNRNPU, HNRNPA3, UBL4A, UBD, UBL5, RTF1, ILF3 and NKKB2, was functionally involved in the process of translation, metabolism of proteins, intrinsic pathway for apoptosis, viral mRNA translation, membrane trafficking, cell cycle and apoptosis. The green module (IM3) containing genes like UBC, CHD3, HTT; RAD21 and PTGES2, was engaged in biological function like activation of matrix metalloproteinases, meiosis, chromosome maintenance and extracellular matrix organization.

### Functional network of adaptive immune transcripts

**Fig 9:**
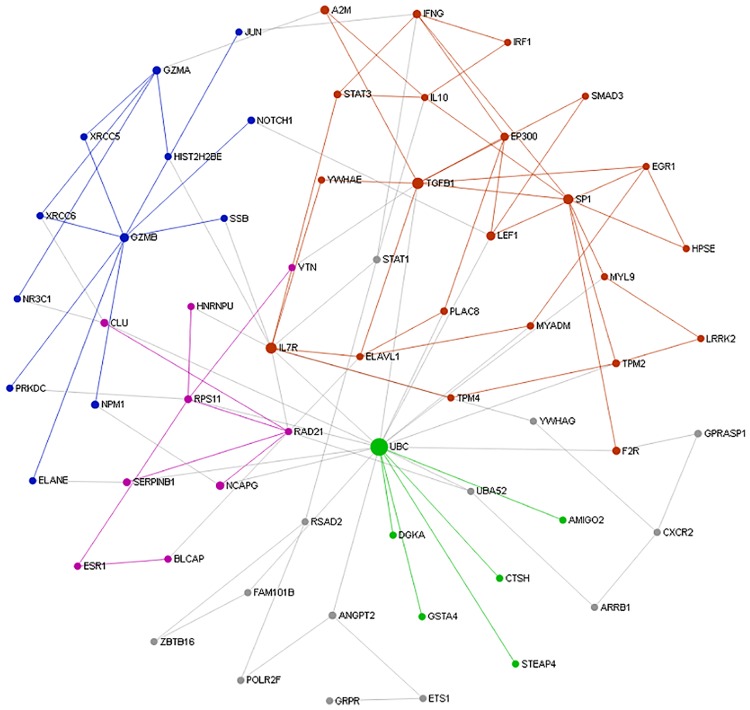
Network of PRRSV vaccine induced adaptive immune transcriptome in PBMCs. The picture depicts the interconnected network of PRRSV vaccine induced differentially expressed genes in PBMCs at 28 days after PRRSV vaccination compared to before vaccination in Pietrain pigs. Each circle indicates the node or member genes of the network. The diameter of the circle corresponds to the values of two centrality measures that is degree and betweenness of particular node. The larger diameter indicates the higher potential of the nodes to be the hub genes of the network. The network modules with corresponding genes are indicated by different colors (purple: AM0, blue: AM1, pink: AM2, green: AM3 and yellow: AM4).

The sub-network enrichment analysis of PRRSV vaccine induced adaptive immune transcripts in PBMCs (Fig 9) identified TGFß1, IL7R, RAD21 and GZMB as highly interconnected genes, and are likely to be the potential hubs of the functional network. The purple module (AM0) containing genes ILR7, TGFß1, SP1, IL-10, EP300, IFNG, EGR1, STAT3, TPM2, LEF1, IRF1 and are biologically linked to cytokine signaling in immune system, signaling by TGF-beta receptor complex, influenza virus induced apoptosis and signaling to STAT3. The blue module (AM1)containing the gene GZMA, GZMB, HIST2H2BE, XRCC6, XRCC6 and JUN, was linked to biological function of HIV infection, disease, DNA repair, integration of provirus and nucleosome assembly. The pink module (AM2) containing genes such RAD21, RPS11, ESR1, CLU and HNRNPU, was involved with biological process like cohesin loading onto chromatin, M phase and mitotic prometaphase. The green module (AM3) containing genes like UBC, CTSH, DGKA, STEAP4 and AMIGO, was engaged in biological function like glutathione conjugation, MHC class II antigen presentation and adaptive immune system. Yellow module (AM4) containing genes GPRASP1, UBA52, ARRB1, CXCR2 and YWHAG, was functionally involved with NF-kB activating and signal survival, assembly of HIV virion, STING mediated induction of type 1 IFN, signaling by NOTCH and apoptosis.

### The RT-qPCR validation

**Fig 10:**
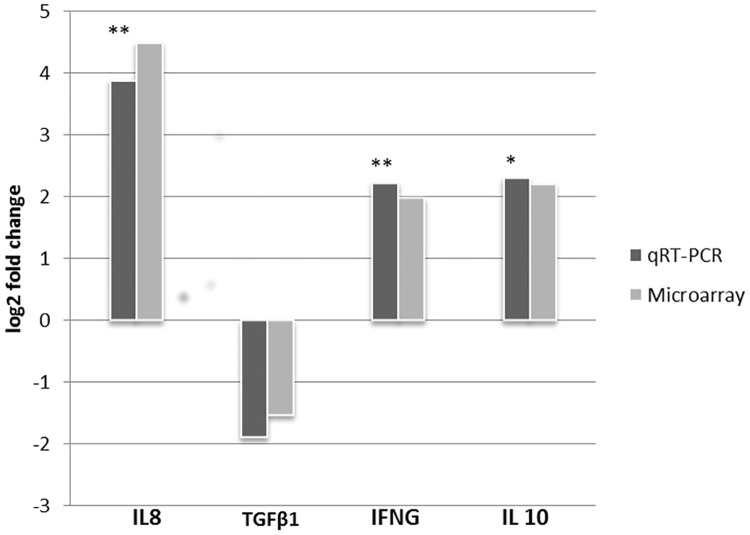
The RT-qPCR validation of the microarray data. The Y-axis of the bars indicates the log2 fold changes of each gene in PBMCs collected at one day after vaccination compared to control determined by RT-qPCR and microarray. Asterisk mark indicate the level of statistical significance (* <0.05; ** <0.01).

To validate the expression level of genes estimated by microarray, four differentially expressed genes such as IFNG, TGFß1, IL-8 and IL-10 were selected for real time qPCR analysis. The expressions of all selected genes obtained from microarray and the qPCR are presented in Fig 10. The RT-qPCR expression values of all genes were aligned with the microarray data with a high correlation (pearson correlation coefficient, r ≥ 0.929; *P* ≤ 0.0514).

## Discussion

Transcriptome profiling of PBMCs is receiving more interest in evaluating host immune response to infectious diseases, since PBMCs play central role in immune system. PBMCs are a heterogeneous population of blood cells that include monocytes, lymphocytes (T cells, B cells and NK cells) and dendritic cells. These blood cells patrol through entire body systems and immediately respond to both internal and external stimuli. Researches have shown that porcine PBMCs can display gene expression patterns which are characteristics for certain pathogenic infection, for instance, classical swine fever [41] and tetanus toxoid [42]. In the current study, whole transcriptome profiling of PBMCs was performed in three individual piglets to characterize the gene expression changes associated with the innate as well as the adaptive immune response to PRRSV vaccine in Pietrain pigs. One of the limitations of this study is the use of low number of biological replicates, since a higher number of replicates in the microarray experiment would leads more statistical power, thereby more robust results. Nevertheless, several other groups like ours, have implemented three biological replications in global gene expression studies to characterize the host-PRRSV interactions ([15, 18–20]). We compared the global transcriptome profiles of PBMCs collected at day one (D1) and day 28 (D28) post PRRSV vaccination with that of before vaccination (D0) from the same pigs. In a similar design, transcriptome profiles of pre infected (0h) whole blood samples were compared with that of repeatedly collected post infected samples from the same individuals to characterize the immune responses to PRRSV [22].

The current study yielded a transcriptome dataset comprised of 411 differentially expressed genes in PBMCs after PRRSV vaccination. The robustness of this dataset was confirmed through measuring the expression levels of four selected differentially expressed genes in the same sample by RT-qPCR (Fig 10). Differential gene expression analysis showed that significant changes in PBMCs transcriptome profiles occurred at day one post PRRSV vaccination in Pietrain pig. The proportion of down regulated genes was higher than the upregulated genes for both contrast pairs (Table 2). The exact mechanism of this global down regulation is yet to be clarified, however, we speculated that this may be attributed by the host genetics. Because, host factors like age [25] and breed [19] in particular, have strong influence on the development of immunity against PRRSV. An aberrant host immune response characterized by the consistent down regulated genes was reported in the PRRSV infected alveolar macrophages of pigs [18]. In the same line, our recent RNA-seq analysis also revealed the global down regulation of altered transcripts in the PRRSV infected lung dendritic cells obtained from Pietrain pigs (Proll et al. 2016, unpublished). Surprisingly, the gene ontology and pathway analysis revealed a central role in the early vaccine response for genes those are involved in pro-inflammatory responses via cytokine-cytokine receptor signaling pathway, CD28 dependent VAV1 pathway and signaling by interleukins. Over expression of IL8 and CCR7 indicated that PRRSV vaccine is able to induce a proinflammatory response in PBMCs. The development of anti-viral innate immunity launches through sensing the viral protein or nucleic acid by the so-called specific receptor, the pathogen recognition receptors (PRRs), expressed constitutively in the host immune cells [43], which in turn induce the proinflammatory response [44]. After intramuscular vaccination, vaccine antigen can reach the blood circulation through bypassing the pulmonary alveolar macrophages, where the cytopathic replication takes place. Our results are in line with findings of a recent meta-analysis performed to characterize PRRSV specific immunity from published transcriptome studies [45]. The meta-analysis showed that the differential expression of a cell surface receptor involved in cytokine regulation, TREM1, along with inflammatory responses toll-like receptor genes TLR2, TLR4, cytokines including IL-1b, IL6 and IL18 and chemokine including CCL2 and CCL3 were involved with PRRSV specific host responses [43]. RNA-seq analyses of transcriptome profiles of PRRSV infected porcine tracheobronchial lymphnodes [46] and lung tissue [47] also revealed that PRRSV induces proinflammatory response.

The interferon response is an well-known innate immune reaction developed upon virus infection or vaccination. We observed an overexpression of IFNG at 28 days post vaccination but not at 1 day post vaccination, which signals a delayed induction of innate anti-viral immunity to PRRSV in PBMCs. This finding is in line with previous studies, where several microarray experiments reported a dampened expression of type I IFN response during PRRSV infection indicating an inadequate stimulation of the innate anti-viral immunity [19, 47, 48]. Similarly, a gradual development of the interferon-gamma response to PRRSV infection has been reported in pig [2]. In contrast, a strong elevation of IFN-ß at 9 h post infection but a slightly elevated expression of IFN*α* was observed in alveolar macrophage infected with PRRSV [18]. However, Zhang and colleagues stated that PRRSV does not fail to induce IFN*α* or IFN-ß mRNA expression in monocyte derived dendritic cells, but protein seems to be blocked post-transcriptionally [49] which demands the investigation of potential role of post transcriptional regulators like miRNAs in PRRSV induced IFN responses.

Transcription factors (TFs) are regulators of gene expression. In mammalian genome, genes are usually in a default “off” state and TFs serve mainly to turn gene expression “on” through recognizing specific cis-regulatory DNA sequences at the promoter regions of target genes [50]. The current analysis revealed the involvement of transcription factors including 120-kDa CRA-binding protein, E4F1, NF1, Tel-2a, HEB and NRF-2 with PRRSV vaccine mediated innate immunity; and ONECTU1, SMAD1 and MYC with adaptive immunity in PBMCs (Fig 6). A total of 27 differentially expressed genes were under regulation of these seven transcription factors identified in PBMCs of PRRSV vaccinated pigs (Table 4), many of altered genes have already been linked to host-PRRSV interaction [15, 17, 18]. Among the TFs identified in this study, MYC has been previously reported to be involved with the swine host response to PRRSV infection [45]. We identified ANGPT2, IFNG, PLAC8 and TGFB1 as potential target genes of MYC transcription factor. The MYC regulates the expression of two immune checkpoint proteins on the tumor cell surface, the innate immune regulator, CD47 (Cluster of Differentiation 47) and the adaptive immune checkpoint, PD-L1 (programmed death-ligand 1) [51], thereby initiates and maintains the tumorigenesis. The involvement of some other transcription factots like interferon regulatory factors (IRF1, IRF3, IRF5 and IRF8), HMGB1, NFkB, EGR1, BCL3, PYCARD MYCN and NFE2L2 in the transcriptional mechanism of immune response to PRRSV in pig has been identified through a meta-analysis [45]. The actions of transcription factors regulate the unique expression of each gene in the different cell types during development process.

The cellular sub-population of PBMCs may have individual roles on development of vaccine immunity. The cell type enrichment analysis revealed that differentially expressed genes specifically expressed in CD4+ T cells, CD8+ T cells, CD14+ and CD33+ monocytes during early stage; and lymphnode, thymus, BDCA4+ dendritic cells, CD4+ T cells and CD8+ T cells in later stage of vaccine immunity (Fig 7). This could indicate that the expression patterns of the genes were not solely due to transcriptional changes but possibly also due to a difference in demographics of PBMCs subsets recruited into the blood. Shimizu et al. [52] observed a remarkable decrease in CD4+ T cells after 3 days PRRSV infection in pigs; this study also reported slight decreases in CD8+ T cells at 3 dpi, followed by substantially increased levels [52], while at the same time, the ratios of CD4+/CD8+ T cells were significantly lower between day 3 and 28 post-inoculation compared with that of day 0 [52]. However, the proportion of CD4+ and CD8+ T cells were found to be significantly decreased for a few days shortly after PRRSV infection, but returned to pre-infection levels on 8–10 days post infection [53]. Renukaradhya et al. [54] performed a comprehensive analysis of innate and adaptive immune responses in dual-virus infected pigs and reported that reduced innate NK-cells population along with increased frequencies of CD4+ T cell, CD8+ T cells and myeloid cells resulted from PRRSV infection in pigs. The PRRSV infection is reported to causes an increase in CD14+ expression throughout the early stage of infection, due to a rise in CD14+ monocytes that differentiate to macrophages and migrate to bronchoalveolar spaces [55]. Silva-Campa et al. [56] observed that PRRSV infection increases the frequency of T cell regulatory cells (Tregs) with the phenotype CD4+, CD8+, CD25+ and Foxp3^*high*^. Therefore, this information on cell-type specific gene expression patterns associated with PRRSV vaccine immunity could be an important add-on for the PRRS research.

Innate immune response traits like other quantitative traits, are not regulated by straightforward linear pathways but rather by networks of complex molecular interactions [57]. Thereby network analyses based on larger immune-specific gene database [37] proved to be a more effective strategy for the identification of genes that regulate the immune response to PRRSV vaccine in PBMCs. Among the hub genes of the network, the lymphocyte-specific protein tyrosine kinase (LCK) gene was found to be one of the potential hubs of functional network (Fig 8). LCK encodes p56 (LCK), a non receptor protein-tyrosine kinase of the SRC oncogene family that is involved in transduction of T-cell receptor (TCR)-mediated activation of T-cell. The signal transduction cascades are activated following antigen binding to the TCR and in concert with engagement of other co-receptors and their associated ligands (such as CD4 and major histocompatibility complex (MHC) class II, CD28, B7, CD8, and MHC I) [58]. Functional enrichment of the network module revealed that the innate immune transcripts are clustered in four modules participating in four major groups of biological functions. The ubiquitination was found to be a key cellular processes significantly upregulated with the transcriptome alteration in PBMCs at early after PRRSV vaccination. We observed the over expression of ubiquitin gene family such as UBC, UBB and UBL5 in PBMCs after vaccination. Moreover, UBC was found to be as one of the potential hubs of the functional network of PRRSV vaccine induced innate immune transcriptomes in PBMCs. The ubiquitination is a post-translational modification process that has been implicated in the regulation of a wide variety of cellular process. The genetic and biochemical evidence suggest that protein ubiquitination and deubiquitination are of fundamental importance in the regulation of the innate and adaptive immune system [59]. The over-expression of porcine ubiquitin specific protease 18 (USP18) is reported to reduce the in-vitro PRRSV replication by altering the cellular distribution of two subunits of NFkB heterodimers (p56 and p50); [60] which indicates the role of USP18 as a host restriction factor during innate immune response to PRRSV. In a subsequent study, the SNP G-1533A polymorphism in the promoter region of the porcine USP18 gene has been suggested as a potential DNA marker for the resistance to PRRSV [61]. Therefore, the ubiquitination process might influence the transcriptional network of PRRSV vaccine induced innate immune response in PBMCs.

The adaptive immunity is specific to the pathogen and the components of the adaptive immune system are also likely contributing to PRRS resistance in pigs. The sub-network analysis of the current microarray study showed the evidence of adaptive B and T cell immunity developed from PRRSV vaccination in pigs. Though adaptive immunity is likely to be predominated by B cell function, however, T lypmhocytes in parallel also have significant contribution in adaptive immunity [62]. The conjugation of viral antigen to a protein carrier (adjuvant) provides foreign peptide antigens that are presented to the immune system and thus recruit antigen-specific CD4+ Th cells which is referred to as T dependent antibody responses [13]. A hallmark of T-dependent responses of live attenuated viral vaccines, is to induce both higher-affinity antibodies and immune memory along with generation of CD8+ cytotoxic T cells. Down regulation of immunosuppresive cytokine TGFß1, and upregulation of interferon IFNG and chemokine CXCR2, VAV1, SMAD3, GYMA, GYM5 and transcription factor STAT1 were found to be among regulators of the transcriptional network of vaccine induced adaptive immunity. This is consistent with the hypothesis that there are possibilities for association of PRRS resistance genes with the cells of adaptive immunity, namely the T and B cells [63]. Major biological pathways involved were TGF-beta receptor signaling pathways, AP-1 transcription factor network, granzyme mediated apoptosis, NOTCH2 signaling and IL-12 mediated signaling. The perforin-mediated apoptosis is principally regulated by IL-10 secreted from cytotoxic T lymphocytes (CTLs) [62]. Type 1 PRRSV strains have been reported to induce IL-10 production in infected dendritic cells [64].

Induction of neutralizing antibody (NAb) response is a potential indicator for the vaccine-based adaptive immunity. However, the specificity as well as the level of NAb titre may vary and are likely attributed to establishment of protective immunity. Previous studies suggested that a higher level of PRRSV-specific NAb titre (1:8 to 1:32) in blood is required to prevent the subsequent infection [65]. In this study, PRRSV vaccine induced neutralizing antibody titre (S/P ratio) rose around 1:12 at 28 days post vaccination and remain elevated over 42 days post vaccination (Fig 1). This was supported by the findings of Meire et al. [2] and Yoon et al. [66], who reported that serum antibodies with PRRSV-neutralizing activity appear only at periods equal or higher than 28 days post infection. The timing of peak response may vary with type of antibodies, for instance the PRRSV-specific IgM could be detected at 7 days post infection (PI), with titre peaking between 14 and 21 day PI and decreasing to undetectable levels around 40 days PI [67]. The earliest antibodies detected that are directed against the 15kDa N protein which seems to be unable to provide sufficient protection [65]. However, there is a positive correlation between the level of vaccine-induced serum NAb titre and the level of protection against PRRSV infection [68]. Overall, the anamnestic induction of plasma antibody response at day 28 post vaccination was suggestive for the development of adaptive immunity to PRRSV vaccine in the studied piglets. Therefore, it would imply that the gene expression changes in PBMCs at 28 days post primary vaccination may reflect the transcriptional activity associated with adaptive immune response to PRRSV vaccination in Pietrain pigs.

## Conclusions

This study support a model in which PBMCs transcriptome alterations are involved in upregulation of CD28 dependent VAV1 pathway, signaling by interleukins and ubiquitination pathway at the initial 24 hours after vaccination; and upregulation of IL12-mediated signaling events, AP-1 transcription factor network and TGF-beta receptor signaling pathways at 28 days after PRRSV vaccination in pigs. Network analysis sorted out the potential regulatory genes involved with induction of innate immune response and subsequently contributes to the development of adaptive immune response in PBMCs to PRRSV vaccination. Among the vaccine induced genes, LCK, STAT3, ATP5B, UBB and RSP17 were found to be the potential candidates for innate immune responses to PRRSV vaccine in the peripheral blood. Further work is required to determine whether polymorphisms linked to genes identified in this study affect the innate immune response trait in pig populations immunized with PRRSV vaccine.

At 28 days post PRRSV vaccination, a plateaued antibody response was observed in plasma, concomitantly significant transcripts abundance was identified by microarray analysis in PBMCs. Among the differentially expressed genes, TGFß1, IL7R, RAD21, SP1 and GZMB were highly interconnected hub genes of functional network, thereby likely to be the potential candidates to predict the PRRSV vaccine induced adaptive immune response in the peripheral blood. The degree of association between the antibody response and the transcriptome alteration induced by PRRSV vaccine could further be tested through expression of these adaptive response candidates in the PBMCs of pigs with extreme antibody response phenotype in a larger population.

## Supporting information

S1 Table DEGs associated with innate immunity. List of differentially expressed genes in porcine PBMCs associated with innate response to PRRSV vaccine.
(XLSX)

S2 Table DEGs associated with adaptive immunity. List of differentially expressed genes in porcine PBMCs associated with adaptive response to PRRSV vaccine.
(XLSX)
